# Use of Machine Learning Classifiers and Sensor Data to Detect Neurological Deficit in Stroke Patients

**DOI:** 10.2196/jmir.7092

**Published:** 2017-04-18

**Authors:** Eunjeong Park, Hyuk-Jae Chang, Hyo Suk Nam

**Affiliations:** ^1^ Cardiovascular Research Institute Yonsei University College of Medicine Seoul Republic Of Korea; ^2^ Department of Cardiology Yonsei University College of Medicine Seoul Republic Of Korea; ^3^ Department of Neurology Yonsei University College of Medicine Seoul Republic Of Korea

**Keywords:** medical informatics, machine learning, motor, neurological examination, stroke

## Abstract

**Background:**

The pronator drift test (PDT), a neurological examination, is widely used in clinics to measure motor weakness of stroke patients.

**Objective:**

The aim of this study was to develop a PDT tool with machine learning classifiers to detect stroke symptoms based on quantification of proximal arm weakness using inertial sensors and signal processing.

**Methods:**

We extracted features of drift and pronation from accelerometer signals of wearable devices on the inner wrists of 16 stroke patients and 10 healthy controls. Signal processing and feature selection approach were applied to discriminate PDT features used to classify stroke patients. A series of machine learning techniques, namely support vector machine (SVM), radial basis function network (RBFN), and random forest (RF), were implemented to discriminate stroke patients from controls with leave-one-out cross-validation.

**Results:**

Signal processing by the PDT tool extracted a total of 12 PDT features from sensors. Feature selection abstracted the major attributes from the 12 PDT features to elucidate the dominant characteristics of proximal weakness of stroke patients using machine learning classification. Our proposed PDT classifiers had an area under the receiver operating characteristic curve (AUC) of .806 (SVM), .769 (RBFN), and .900 (RF) without feature selection, and feature selection improves the AUCs to .913 (SVM), .956 (RBFN), and .975 (RF), representing an average performance enhancement of 15.3%.

**Conclusions:**

Sensors and machine learning methods can reliably detect stroke signs and quantify proximal arm weakness. Our proposed solution will facilitate pervasive monitoring of stroke patients.

## Introduction

Stroke is one of the main causes of death and disability worldwide [1]. One-third of stroke patients experience recurrent strokes. Muscle weakness is the most frequent sign of stroke and is related to disability [2]. Pronator drift, an indication of arm weakness, is mainly caused by subtle upper motor neuron disorders and is measured using the pronator drift test (PDT) [3]. PDT has higher sensitivity than other neurological examinations including forearm roll, segmental motor exam, the Barr test, the Mingazzinis movements, and tendon reflexes [4]. Most stroke patients are diagnosed with the help of trained neurologists who perform bedside neurological examination, including PDT. However, early detection of stroke is critical because the effectiveness of thrombolytic therapy is time-dependent, and earlier treatment results in better outcomes [5]. In addition to the need for instant examination, objectivity and accuracy need to be improved, because the conventional PDT performed by an inexperienced observer can result in missed rapid jitter of arm movement. To improve subjective decision making in the context of the conventional PDT, we developed an objective tool (the iPronator) to measure drift and pronation, and reported its feasibility and usefulness in a previous study [6]. In this study, we propose a decision support solution that can distinguish between the PDT properties of stroke patients and healthy people using representative machine learning algorithms.

## Methods

### Study Design

**Figure 1:**
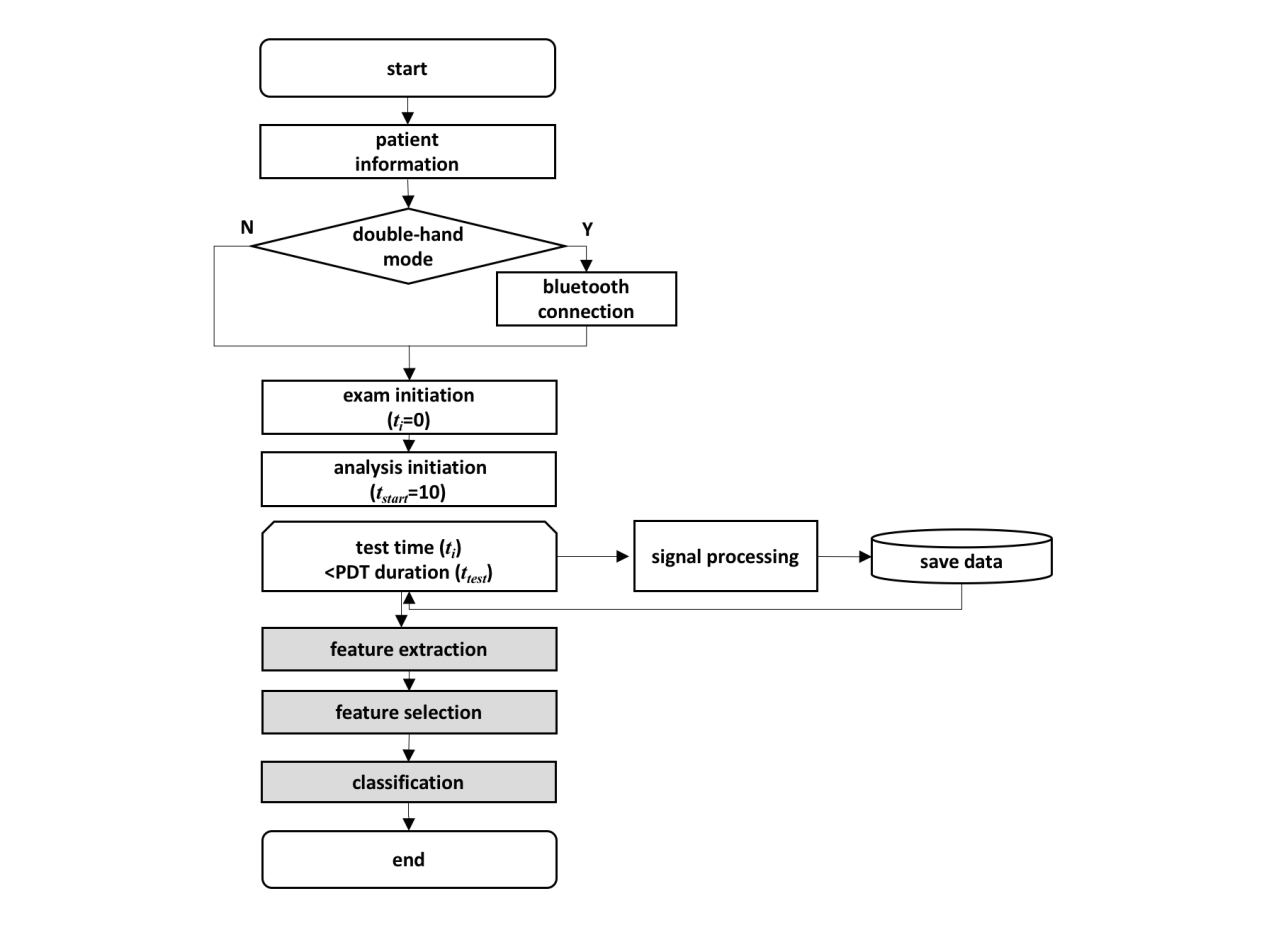
Flowchart of pronator drift test (PDT) software.

We applied machine learning methods to detect arm weakness in stroke patients (Figure 1). First, accelerometer data from PDT were collected from patients and healthy controls for a predefined period. We separated the start time for examination and analysis to exclude the effect of initial dip, which is commonly observed for upper extremity weakness [6]. In this work, the duration of PDT was set to 20 seconds, and the analysis began 10 seconds after the examination started. Next, our feature extraction task produced PDT features from the collected signals. Then, the feature selection task chose effective predictors among extracted features for the enhanced classification. Finally, after feature selection, machine learning algorithms modeled the classification for PDT. This study was approved by the Severance Hospital Institutional Review Board, and informed consent was obtained from all subjects.

### Participants

A total of 26 subjects (10 men and 16 women) were recruited and assigned to the patient group or healthy control group. The ages of the participants ranged from 27 to 84 years, with an average of 58.2 (SD 17.8). During the study period, 16 consecutive stroke patients with mild upper arm weakness were enrolled. Exclusion criteria were patients who were unable to sit and had bilateral arm weakness or preexisting chronic arm weakness. A neurologist graded the muscle power of patients and healthy controls using the Medical Research Council (MRC) scale, which is widely used to evaluate motor weakness (Table 1) [7]. Patients with MRC scores between 0 and 3 were also excluded because PDT was designed for subjects who are able to resist gravity and the weight of the measuring device on the arm. Healthy controls consisted of subjects who had no upper arm weakness and no history of neurological disease. All healthy controls were graded MRC 5. In total, 6 stroke patients were graded MRC 4, 7 were graded MRC 4+, and 3 were graded MRC 5.

**Table 1:** Muscle power grading using the Medical Research Council scale.

Grade	Description
0	No contraction
1	Flicker or trace of contraction
2	Active movement with gravity eliminated
3	Active movement against gravity
4	Active movement against gravity and moderate resistance
4+	Active movement against gravity and strong resistance
5	Normal power

### Sensor Signal Processing

The integrated, low-power, three-axis accelerometer (LIS331DLH, ST-Microelectronics) in the mobile phone was used to measure pronation and drift. The accelerometer has a low-power mode and high accuracy of 1% on its lowest measurement range (±2g) and approximately 0.1% on its highest measurement range (±8g) [8]. Any device equipped with sensors, including mobile phones or wrist bands, can be used as a sensorized PDT tool.

**Figure 2:**
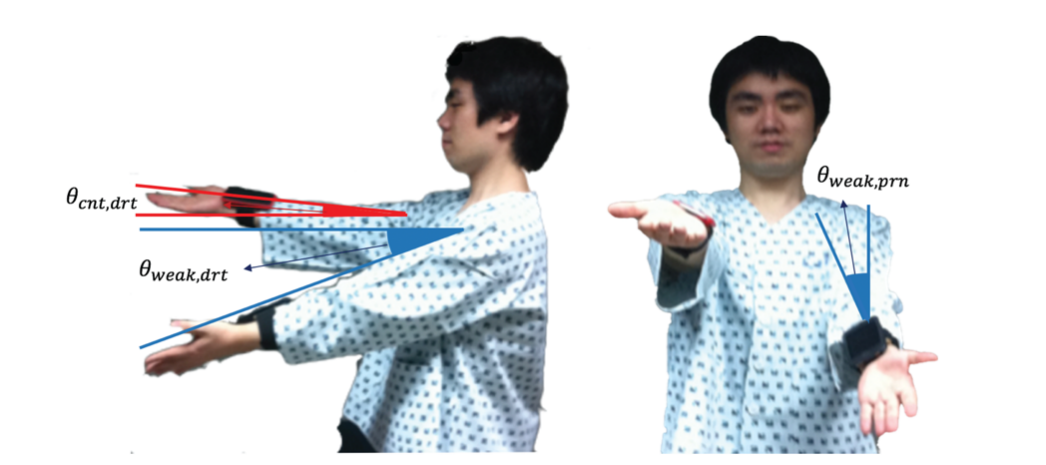
The pronator drift test: (a) the degree of drift in the weak arm and counter-arm of a patient was measured by the drift angle from the horizontal plane, and (b) the degree of pronation was assessed in front of the patient.

Demographic information was collected according to predefined protocol. Patients were asked to conduct the PDT trial after registration. Two sensing devices were placed on each of a subject’s wrists, as shown in Figure 2. When the mode was set to double-hand mode, the two devices were paired with a Bluetooth connection. In the initial state of PDT, patients were asked to extend both arms anteriorly and hold them at shoulder height with palms facing up.

The time frame of the test was initialized, and then the PDT software initiated the measurement of arm movement to calculate the degree of pronation and drift. The procedure continued for the predefined test duration, calculating drift and pronation of the weak side. PDT simultaneously measures the movement of the counter-side by calculating the drift and pronation based on the fact that the counter-limb of the defective side also moves [6,9]. Measured values were subsequently saved for feature extraction in the analysis step. On the basis of collected data for the test duration, the properties of PDT were extracted and input into the classifiers (see Multimedia Appendix 1).

### Decision Support by Pronator Drift Test Classifiers

We performed machine learning classification of PDT results from stroke patients versus those from healthy controls using MATLAB (Mathworks) [10], WEKA (University of Waikato) [11], and Medcalc (Medcalc Software) [12]. As a preprocessing step before machine learning, we implemented feature selection to identify which features were discriminant predictors to enhance the performance of the machine learning algorithms by eliminating redundant and irrelevant attributes [13]. We used a wrapper approach for feature selection; this assesses subsets of extracted features according to their contribution to the classification performance [14,15]. Feature selection considers the employed classification model as an unseen part and assesses the subset of features according to their usefulness to a given classifier. Best-first search was used to traverse the space of candidate subsets and greedily find the optimal subset [15].

Next, a series of machine learning techniques [16], namely support vector machine (SVM), radial basis function network (RBFN), and random forest (RF), were implemented. We selected such methods based on the findings in the research that compared 17 families of classifiers using 121 datasets, resulting in RF, SVM, and neural network–ranked top families [17]. Details of these machine learning algorithms are beyond the scope of this paper; thus, we only provide a brief description of each method.

### Support Vector Machine

**Figure 3:**
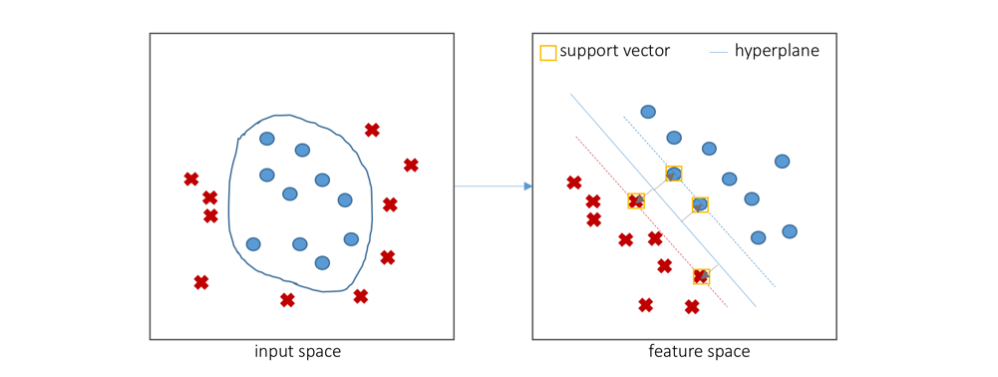
An example of a support vector machine with four support vectors in feature space.

SVM is a machine learning algorithm developed by Cortes and Vapnik [18]. An SVM as a classifier trains a function that calculates a score for a new input to separate samples into two classes by building a hyperplane, which maintains a maximum margin between support vectors (Figure 3).

If the output of the scoring function is negative, then the input is classified as belonging to the negative class; if the score is positive, the input is classified as belonging to the positive class. The scoring function is expressed as (eq.1; see Multimedia Appendix 2), where *x*^(i)^ represents the *i*^th^ input vector, *y*^(i)^ represents the class label of the *i*^th^ training data, and α_i_ is the coefficient associated with the training sample. The function *K*, which is called a kernel function, operates on the two vectors and reduces dimensions to simplify computation of the product of vectors. Among various kernel functions, we applied the polynomial kernel for the PDT classifier [19].

### Radial Basis Function Network

**Figure 4:**
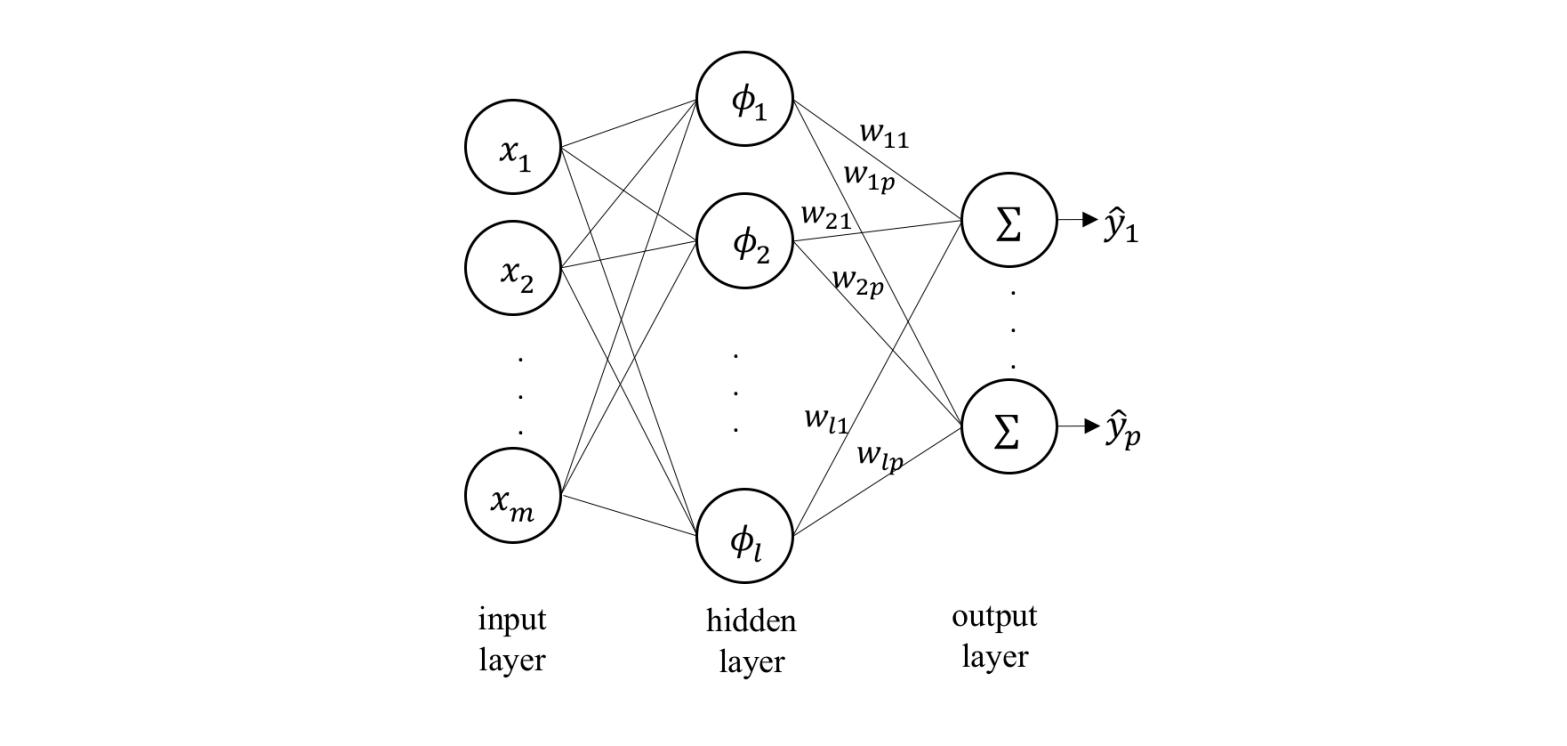
The architecture of an radial basis function network.

RBFN is a neural network classifier that computes the Euclidean distance between a new input vector and the prototype vector corresponding to each neuron to measure the similarity between them [20] (Figure 4).

Each neuron’s activation function is denoted as (eq. 2; see Multimedia Appendix 2), where μ_i_ and β_i_ are the prototype vector and the coefficient of the corresponding neuron *i*, respectively. The training process for an RBFN selects the prototype vector, coefficient for each of the RBF neurons, and the matrix of output weights *w*_ij_ between the RBF neurons and the output node *j*. The decision for each class *j* is decided by (eq. 3).

Various approaches have been proposed to select prototypes from input vectors. We applied *K* means clustering as the base function to select prototypes [21].

### Random Forest

**Figure 5:**
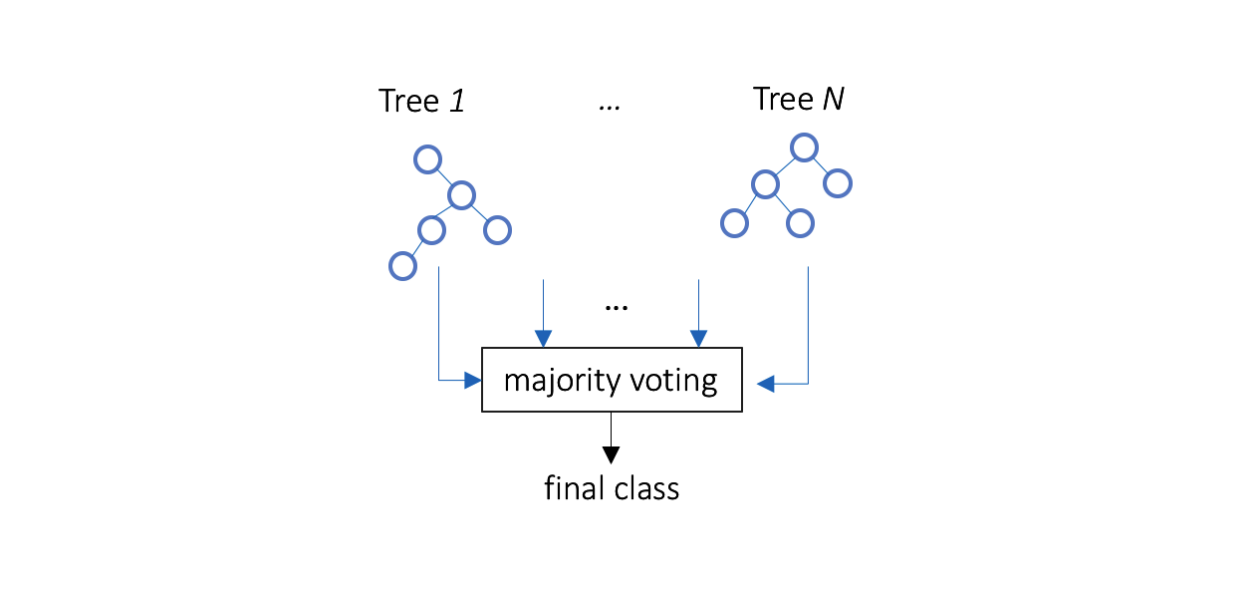
A simplified random forest.

RF is an ensemble predictor that uses a combination of multiple decision trees [22]. Prediction in the training stage is determined by voting from the forest in which an individual tree predicts the target class depending on the values of a random vector sampled independently (Figure 5).

We applied SVM, RBFN, and RF classifiers to the entire set of PDT features. Leave-one-out cross-validation (LOOCV) was applied, because we had a small number of training samples. The performance of classifiers was measured by calculating sensitivity, specificity, the *F* measure, and area under the receiver operating characteristic curve (AUC).

## Results

### Statistical Properties of Pronator Drift Test Features

**Figure 6:**
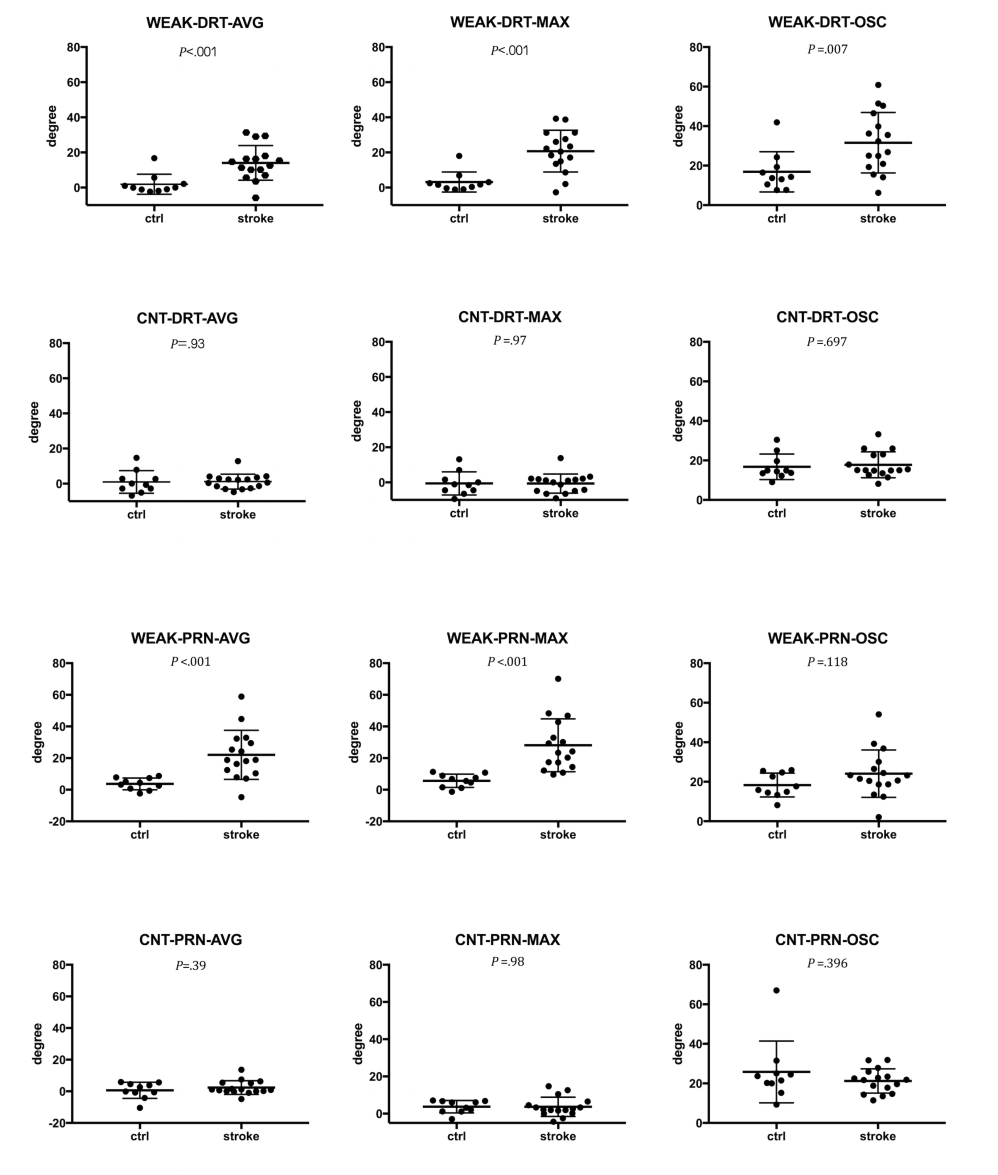
Differences of degree in PDT features between stroke patients and controls. Values are mean, standard deviation, and P value.

We compared PDT features using the *t* test. Figure 6 shows the means and standard deviations of the PDT features. Among the 12 PDT features, WEAK-DRT-AVG, WEAK-DRT-MAX, WEAK-DRT-OSC, WEAK-PRN-AVG, and WEAK-PRN-MAX were significantly different between stroke patients and controls.

### Selected Attributes

Among the extracted PDT features, SVM, RBFN, and RF classifiers selected discriminative features (Table 2). We applied the wrapping approach for feature selection. Feature selection procedure for SVM calculates the usefulness of features and extracts a discriminant feature set {WEAK-PRN-MAX, WEAK-DRT-AVG} for SVM. Feature selection for RBFN reduced all features to three features of pronation on the weak side {WEAK-PRN-MAX, WEAK-PRN-AVG, WEAK-PRN-OSC}. Feature selection for RF resulted in identification of the maximum degree of pronation on the weak side and average drift of the counter-side as useful features for RF classification {WEAK-PRN-MAX, CNT-DRT-AVG}.

**Table 2:** Features selected in pronator drift test classification.

Selected feature	Machine learning classifier			No. of classifiers that selected the feature
	SVM	RBFN	RF	
WEAK-PRN-MAX	X	X	X	3
WEAK-PRN-AVG		X		1
WEAK-PRN-OSC		X		1
WEAK-DRT-AVG	X			1
CNT-DRT-AVG			X	1
No. of features	2	3	2	

### Stroke Classifiers With Selected Pronator Drift Test Predictors

Using the selected features for the SVM classifier, we built an SVM PDT classifier with a polynomial kernel. PDT feature vector *SVM-PDT*_vec_ and score function *f* (*SVM-PDT*_vec_) for the SVM classifier were modeled as (eq. 4; see Multimedia Appendix 2).

**Figure 7:**
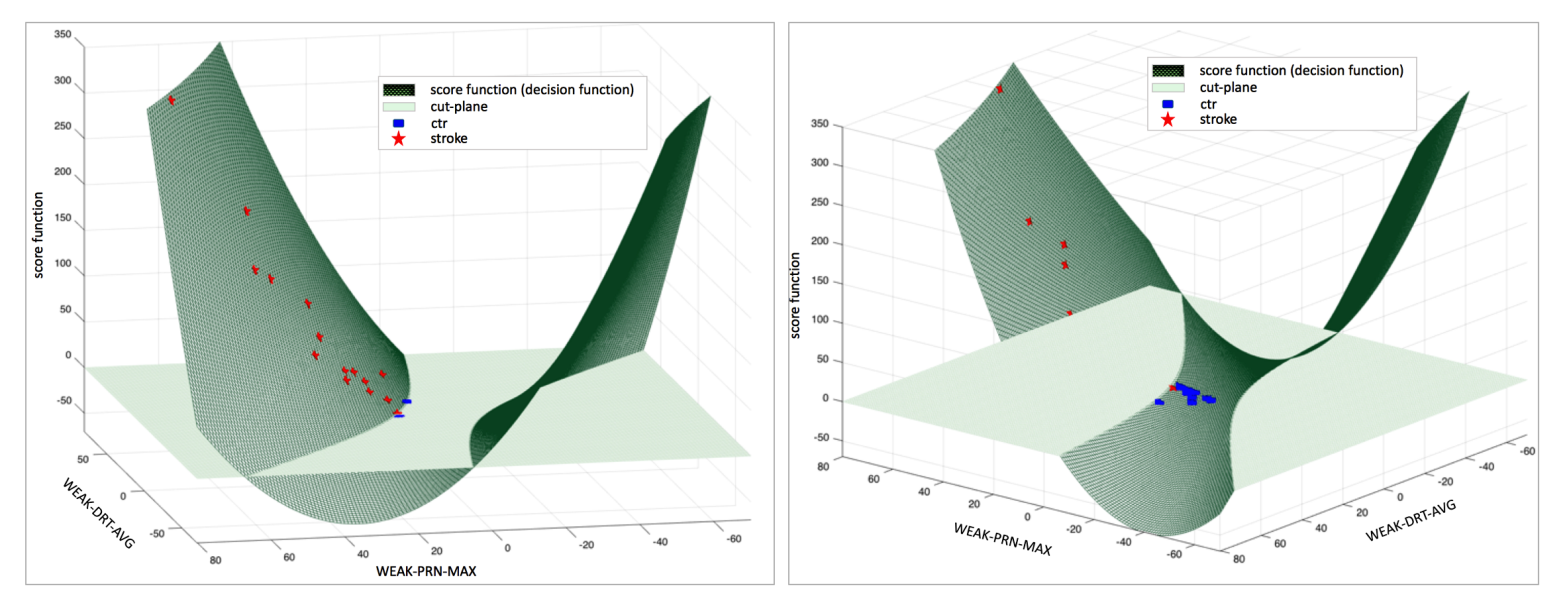
Plot of support vector machine (SVM) score function and decision by the SVM classifier: (a) positive scores of the SVM classifier for input (above the plane); two control cases were misclassified as patients, and (b) negative scores of the SVM classifier for input (below the plane); one stroke patient case was misclassified as a control case.

The derived score function was used to assign training instances into positive class and negative class that contained positive and negative values of the score function, respectively. As shown in Figure 7 (a), stroke patients’ PDT features were mapped on the surface of the score function above the cut-plane. Two control cases were misclassified as belonging to the stroke group. As shown in Figure 7 (b), the score function for the control group produced values less than the cut-plane, and one stroke case was misclassified as a control case.

**Figure 8:**
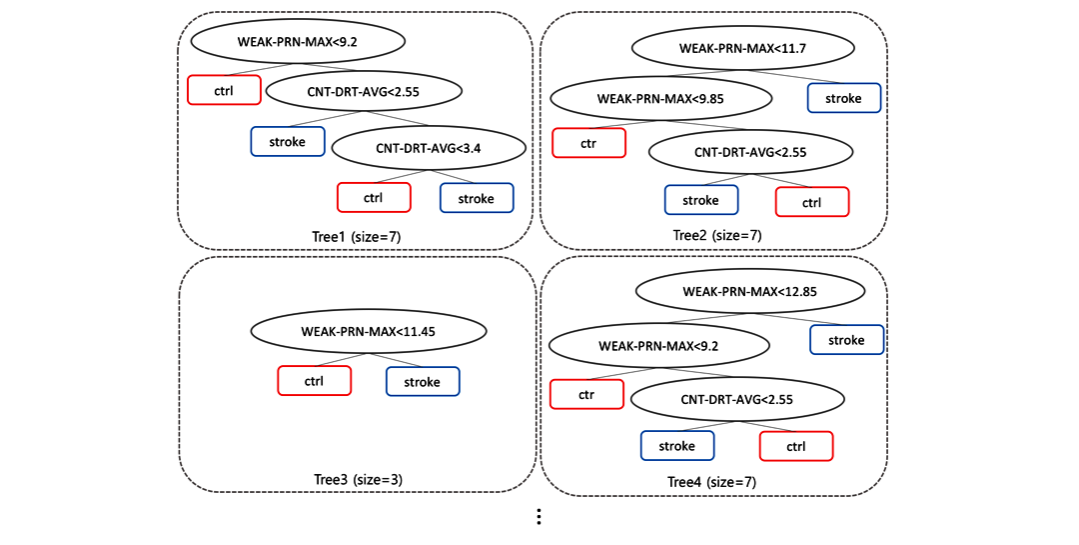
Random forest composed of decision trees as a pronator drift test classifier.

**Figure 9:**
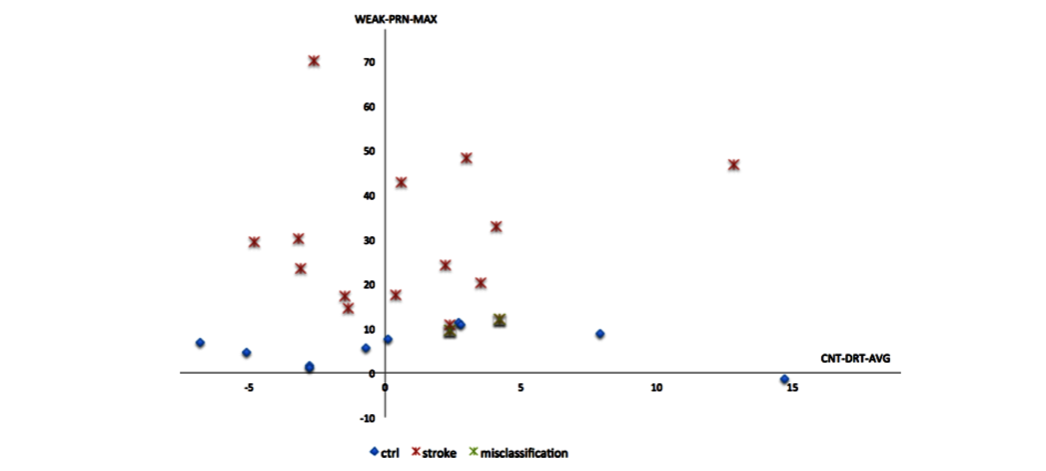
Weakness detection using a random forest, including feature selection.

The RBFN classifier for stroke patients constructed four clusters to calculate radials in the RBFN without feature selection and two clusters for the RBFN including feature selection. The RF classifier combined decision trees as depicted in Figure 8; two cases were misclassified (Figure 9).

### Performance of Stroke Classifiers With Feature Selection

**Figure 10:**
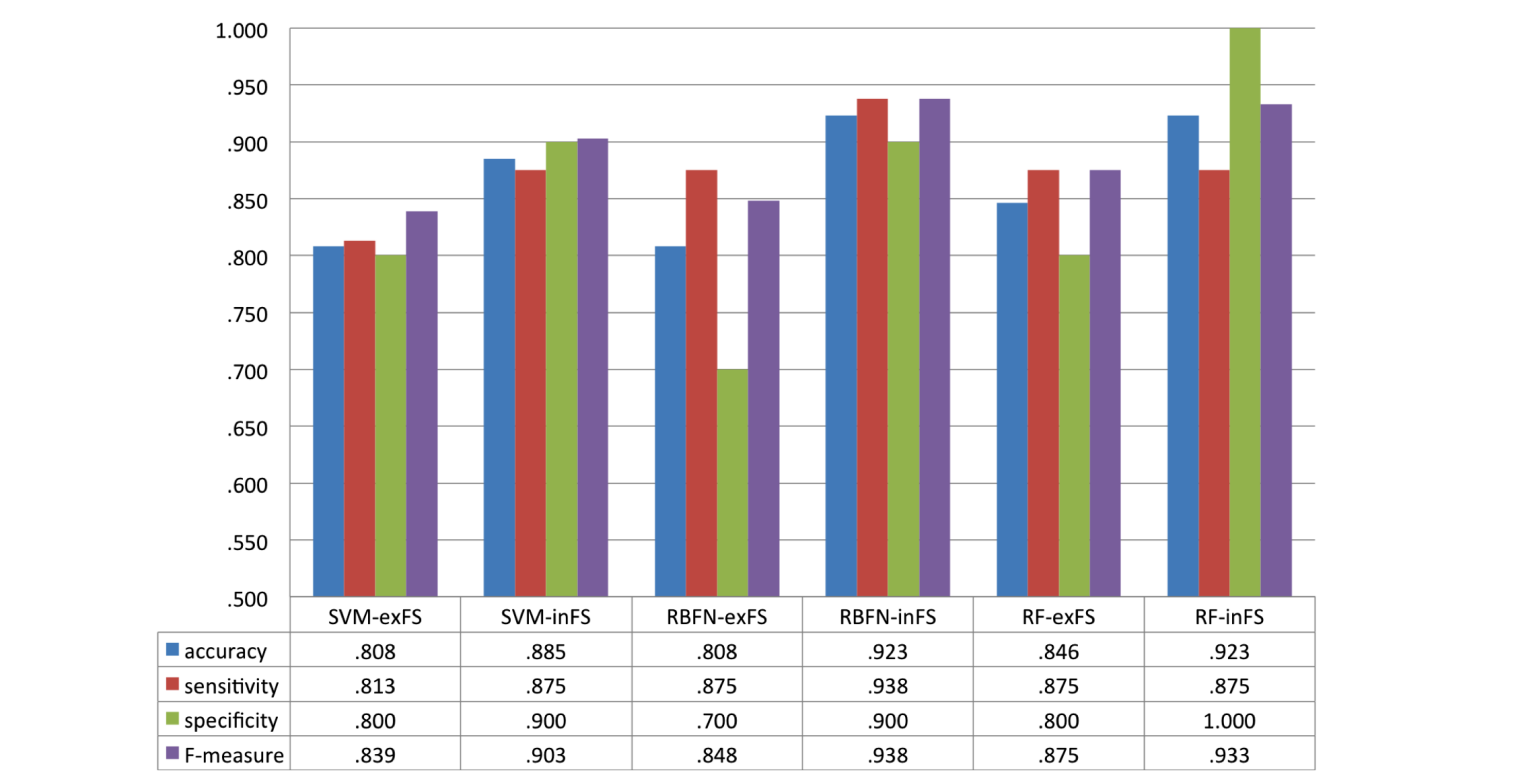
Performance of stroke classifiers excluding/including feature selection.

Figure 10 shows the performance of classifiers in terms of accuracy, sensitivity, specificity, and F-measure. The accuracy of all classifiers was improved by feature selection; accuracies of the classifiers improved by 9.53% (.808 with SVM-exFS [excluding feature selection] vs .885 with SVM-inFS [including feature selection]), 14.23% (.808 with RBFN-exFS vs .923 with RBFN-inFS), and 9.10% (.846 with RF-exFS vs .923 with RF-inFS), respectively.

**Figure 11:**
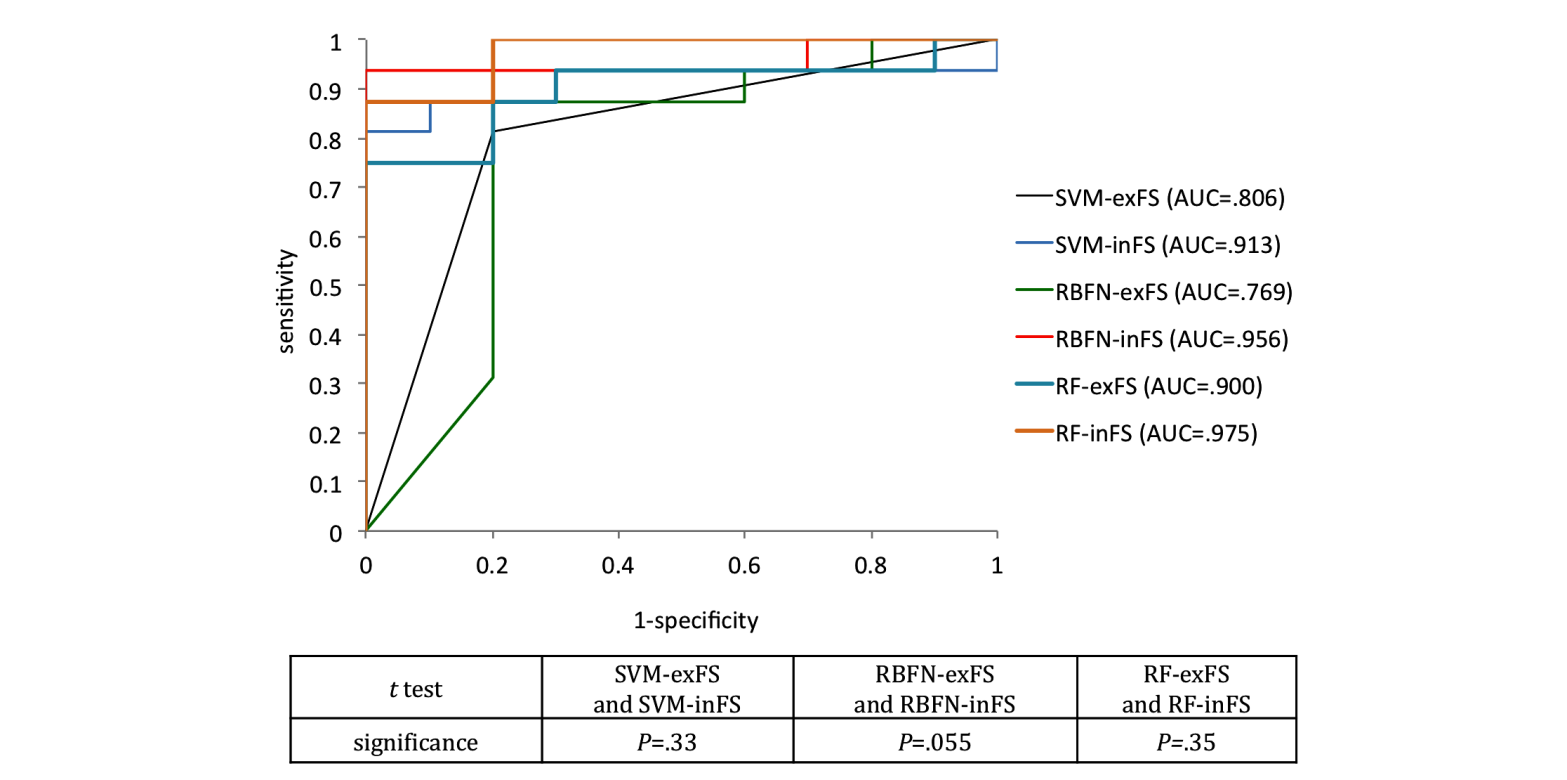
Receiver operating characteristic (ROC) curve of pronator drift test classifiers and t test of area under the receiver operating characteristic (AUC) (SVM-exFS and SVM-inFS: support vector machine excluding and including feature selection; RBFN-exFS and RBFN-inFS: radial basis function excluding and including feature selection; RF-exFS and RF-inFS: random forest excluding and including feature selection).

The stroke classifiers had an accuracy of up to 92.3% for detecting stroke (RBFN-inFS, AUC = .956; RF-inFS, AUC = .975), and RF had the best AUC of .975 when feature selection was applied (Figure 11). To compare the means of the individual AUCs of methods with and without feature selection, *t* test was also performed.

## Discussion

### Summary

We developed a novel method to monitor pronator drift using sensor-equipped devices. We investigated and demonstrated the feasibility of machine learning analysis of the information obtained via the sensors and found that the combination of these methods can detect the neurological deficit of subtle motor weakness. We demonstrated that machine learning–based classifiers correctly classified up to 92.3% of PDT cases.

### Review of Previous Studies

Machine learning has recently been adopted in medicine and its usage includes various medical studies: health care utilization based on patients’ social network data [23,24]; predicting mortality after surgery [25]; estimating the risk of treatment outcomes [26]; predicting deterioration using electronic medical records with physiological signals [27]; and activity monitoring [28]. This popularity is because of the advantage of easily incorporating new data to improve prediction performance [29] and to identify discriminant variables for prediction [30]. Machine learning has also improved assessment and outcome prediction in stroke studies. Decision tree [31], SVM, and neural network [29] have been utilized to predict the outcome of acute ischemic stroke. SVM-integrated regression models have also been proposed to predict stroke [32].

In addition to machine learning analysis, sensor-based measurement improved the detection of abnormality and outcome prediction. Task-oriented, arm-hand training using sensor measurement was introduced in [33], and a machine learning method with pressure sensor–embedded smart shoes discriminated the alcohol-induced gait [34].

In this study, we utilized an off-the-shelf smart device embedding accelerometer for the measurement of arm weakness. The use of mobile phones or general activity trackers elaborated the high accessibility of users. Recent studies demonstrated the validity of using accelerometer in iPhone for the physical activity monitoring [28], the extraction of heart rate [35], and applications for Parkinson and Holmes tremor [36].

We previously developed a sensor-based mobile tool (the iPronator) and reported that the iPronator app was useful and feasible for detecting mild arm weakness and quantifying the degree of weakness. Moreover, the iPronator can also detect functional recovery after one week in patients with acute stroke [6]. In this study, we further evaluated whether machine learning could improve detection of the presence of mild arm weakness after stroke. Although information technology and mobile devices are increasingly used in the management of stroke [37], most researchers have focused on analyzing medical records, including laboratory results, to predict mortality and the outcome of care. However, as far as we know, no studies have reported using machine learning–based classifiers to detect weakness associated with stroke and PDT.

### Predictors for Stroke Decision Support

PDT is known to be a sensitive neurological test of weakness. If a patient has pronator drift, positive test indicates the damage in motor pathway from the opposite side of the brain [38]. The pronator drift is determined by various conditions including motor deficit, sensory deficit, cerebellar drift, parietal lobe lesions, and conversion disorders: cerebellar disease causes outward and upward drift; patients with parietal lobe lesions exhibits loss of position sense, which causes updrift with the involved arm rising overhead; and functional upper limb paresis causes drift without pronation [39]. Due to such various causes of pronator drift, the result of PDT varies on the condition of patients: one study showed that patients with subtle difficulty in routine activity had positive PDT in 38 (76%) out of 50 patients [40], whereas another study showed positive PDT in only 43.8% of patients with cerebral lesions [41]. We infer that such variability originated the outperformance of machine learning methods in the classification of PDT, since machine learning is strong in sophisticated pattern recognition by delineating patterns from relations between less significant variables as well as key variables. As shown in Figure 6 and Table 2, the key variable WEAK-PRN-MAX, which showed a significant difference between patients and controls in statistical analysis, was the dominant feature selected by all classifiers and can therefore be considered a dominant predictor for detecting weakness. In addition, machine learning methods (RF and RBFN) utilized CNT-DRT-AVG and WEAK-PRN-AVG resulting in high detection rate, although both WEAK-PRN-OSC (*P*=.12) and CNT-DRT-AVG (*P*=.93) were not significantly different between the patient and control groups statistically.

In searching for optimal subset of features for classifiers, the wrapper method resolves the problem of high-dimensional features space and feature redundancy to improve the intelligent decision [42]. In [43], feature selection of RF and SVM conducted the phenotyping through limiting the number of variables based on the importance in RF. Especially, feature selection was prominent in the classification with insufficient sample data by restricting the number of features in the classifier to ⌈n/10⌉ for the best performance [44]. In this study, 12 PDT features extracted from sensor signal processing were narrowed down to two or three features to support decision for stroke. We investigated the effect of reduction of dominant features by comparing the performance of stroke classifiers including and excluding feature selection. As shown in Figure 10, the accuracy of RF-exFS classifier (accuracy = .846) obtained higher accuracy than SVM-exFS and RBFN-exFS (accuracy = .808). The result is induced from the RF’s intrinsic property that RF contains the feature selection mechanism in the classification as it randomly selects different variables to construct each tree within its forest. This randomization is known to be effective in eliminating noises and reflecting multivariate interactions with other variables [22]. Therefore, the effect of feature selection appears stronger in RBFN and SVM than RF as shown in Figures 10 and 11. Feature selection improved accuracy by 14.23%, 9.53%, and 9.1% in RBFN, SVM, and RF, respectively. AUC was also improved along with FS by 15.3 % in average (SVM: .806-.913, RBFN: .769-.956, and RF: .900-.975). We conducted the *t* test between AUCs of classifiers with and without feature selection and the difference between RBFN-inFS and RBFN-exFS was most significant (*P*=.06).

### Implications of This Study and Perspectives

Although many mobile devices using sensors have been developed and marketed to doctors and health care providers for years, adoption of machine learning in stroke patients is still in its infancy. In particular, patients or stroke witnesses do not have any tools to detect stroke or communicate with health care providers.

As we described, time is critical in acute stroke management, including thrombolytic treatments. Thrombolytic treatment should begin within 4.5 hours after the onset of a stroke. Moreover, earlier treatment results in better outcomes within the treatment window. Therefore, rapid evaluation of motor weakness is important. To reduce hospital delay and efficiently dispatch patients in emergent medical services, integration of machine learning methods with mobile devices with sensors might be useful.

In addition, evaluation by neurologists may be delayed in busy emergency room. To overcome these limitations and improve patients’ care, a simple bedside tool and objectifying the results are important. The proposed solution can connect patients and health care providers in rapid communication and, ultimately, these approaches may improve the care of stroke patients at low cost.

As another application, the proposed tool might be helpful in monitoring of stroke recurrence in subacute-to-chronic period after stroke. Although we previously demonstrated that the objective of PDT was useful in detecting functional recovery in patients with acute stroke, further long-term follow-up studies can provide its usefulness in detecting stroke occurrence, because machine learning model can be improved with the big data, and personalized history of measurement can provide tailoring in stroke management.

### Limitations and Future Works

In this study, a total of 26 sample data were analyzed by machine learning methods. The performance of machine learning algorithms is known to be affected by the quality and quantity of training data. We adopted LOOCV to complement the small number of instances, and the large data accumulation in further study may diminish the requirement of LOOCV, which requires more computing time and resources.

We also plan to develop a new version of iPronator with small-sized, 3-axis accelerometer and 3-axis gyroscope, since the weight of smart devices may affect the result of PDT. In this study, we excluded the initial dip caused by the mobile phone’s own weight.

The diverse causes of pronator drift can be another limitation for this tool in the detection of stroke, because there exist false positive signs in PDT caused by other lesions outside the motor pathway. The future development extends the current binary classification into multi-classification clustering various causes of PDT.

## Appendix

Multimedia Appendix 1 Features extracted from the pronator drift test.

Multimedia Appendix 2 Equations for pronator drift test classifiers.(eq.1): score function of support vector machine; (eq.2): activation function of neurons in radial basis function network; (eq.3): decision function of radial basis function network; (eq.4): feature vector and score function of support vector machine for pronator drift test.

## Abbreviations

AUC: area under the receiver operating characteristic
FS: feature selection
MRC: Medical Research Council
PDT: pronator-drift test
RBFN: radial basis function network
RF: random forest
SVM: support vector machine
