# Supplementary figures and images for: Use of Machine Learning Classifiers and Sensor Data to Detect Neurological Deficit in Stroke Patients
Source: J Med Internet Res. 2017 Apr 18;19(4):e120. doi: 10.2196/jmir.7092 (PMC5413803; doi:10.2196/jmir.7092)

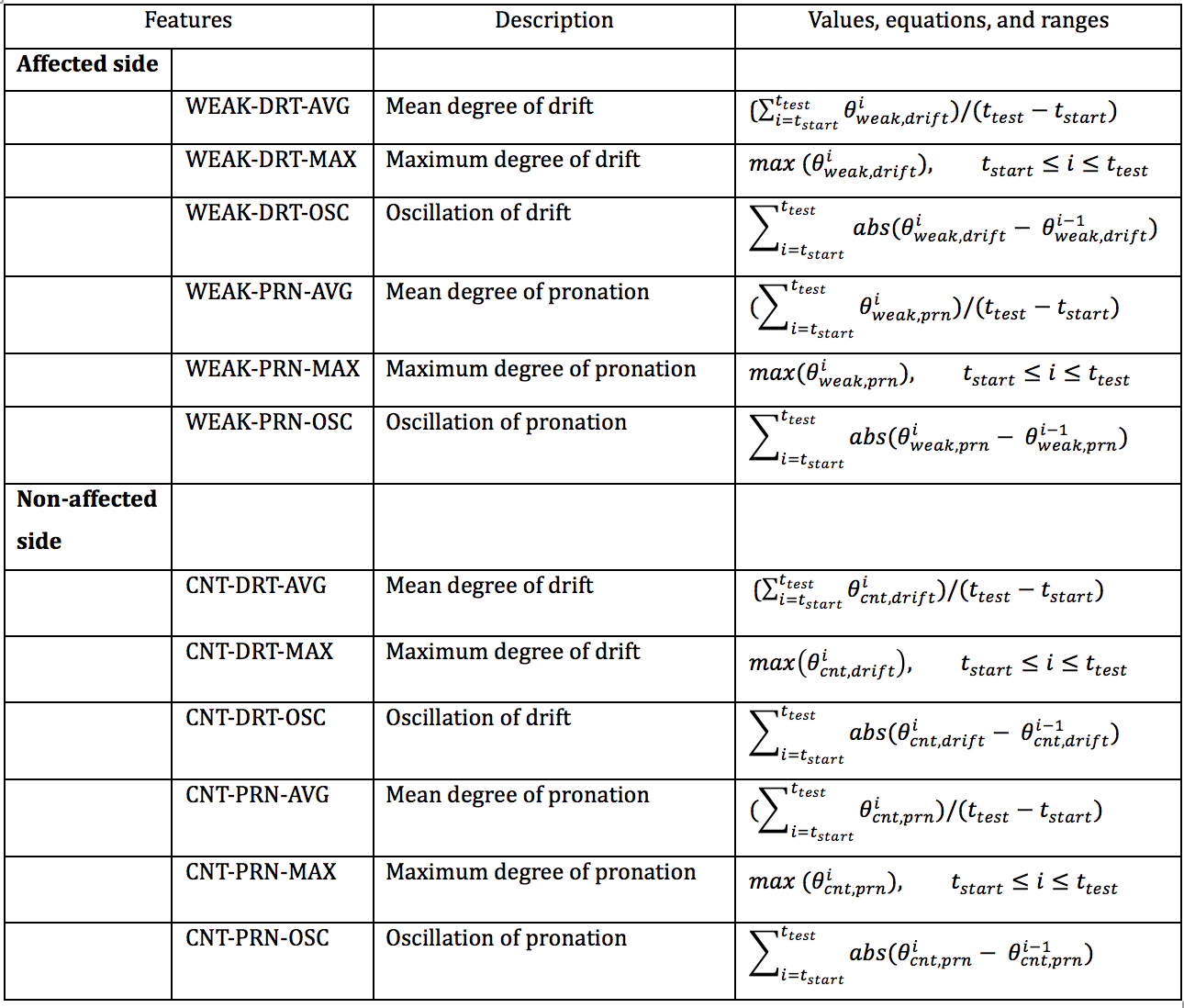

Supplement: Multimedia Appendix 1 [file jmir_v19i4e120_app1.png]

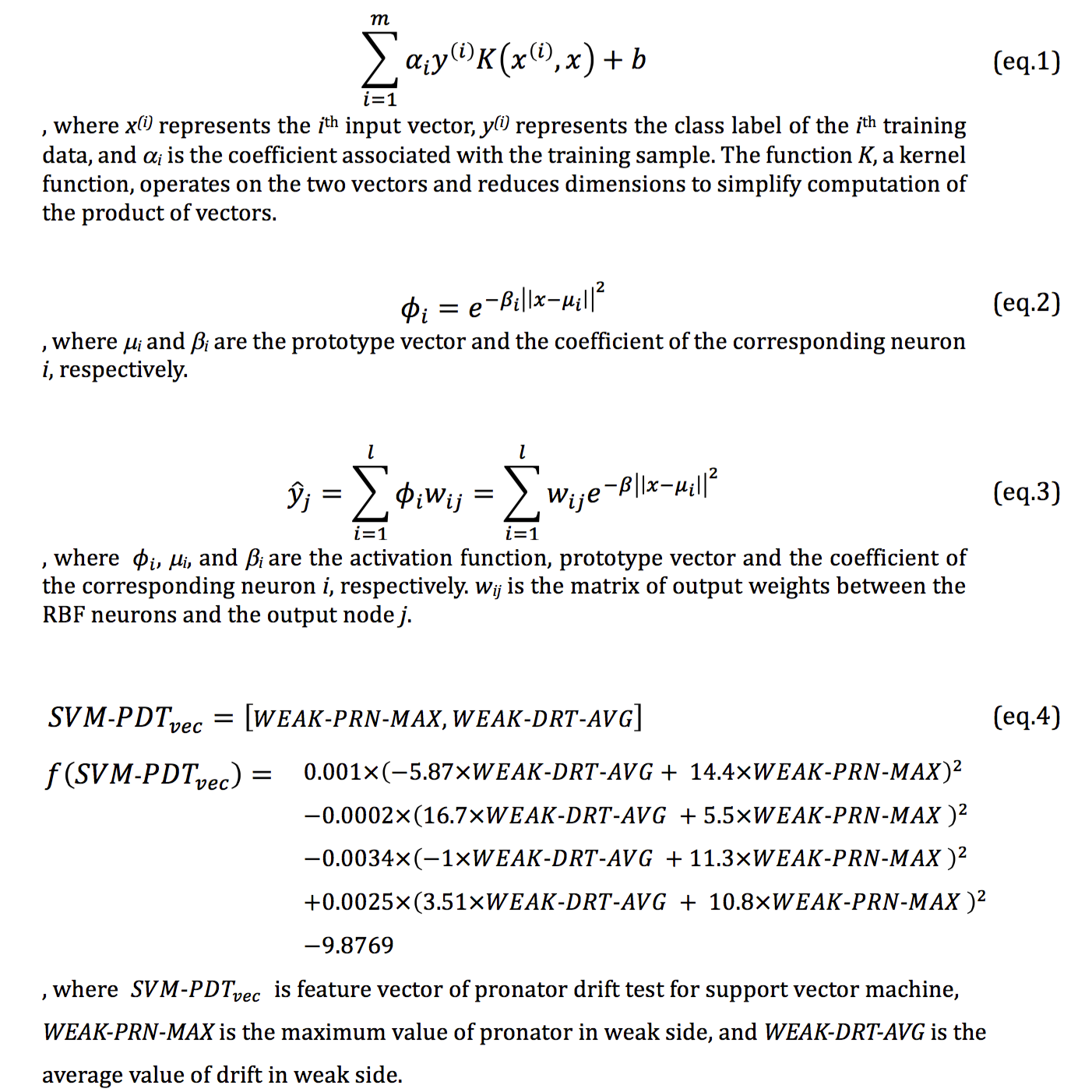

Supplement: Multimedia Appendix 2 [file jmir_v19i4e120_app2.png]
